# Ethnicity, religious affiliation and girl-child marriage: a cross-sectional study of nationally representative sample of female adolescents in Nigeria

**DOI:** 10.1186/s12889-020-08714-5

**Published:** 2020-04-29

**Authors:** Jacob Wale Mobolaji, Adesegun O. Fatusi, Sunday A. Adedini

**Affiliations:** 1 Department of Demography and Social Statistics, Obafemi Awolowo University, Ile-Ife, Nigeria; 2 Department of Community Health, College of Health Sciences, Obafemi Awolowo University, Ile-Ife, Nigeria; 3 Academy for Health Development (AHEAD), Ile-Ife, Nigeria; 4 Medical Research Council: Respiratory and Meningeal Pathogens Research Unit, University of the Witwatersrand, Johannesburg, South Africa; 5 Programme in Demography and Population Studies, Schools of Public Health and Social Sciences, University of the Witwatersrand, Johannesburg, South Africa

**Keywords:** Child marriage, Adolescents, Religion, Ethnicity, Female, Nigeria

## Abstract

**Background:**

The persistently high prevalence of girl-child marriage remains a public health and developmental concern in Nigeria. Despite global campaign against the practice and policy efforts by Nigerian government, the prevalence remains unabated. This study investigates the prevalence and the influence of ethnicity and religious affiliation on the girl-child marriage among female adolescents in Nigeria.

**Methods:**

Data of 7804 girls aged 15–19 years extracted from the 2013 Nigeria Demographic and Health Survey were used. Ethnic groups were classified into five: major Northern ethnic group (Hausa/Fulani); Northern ethnic minorities; two major Southern ethnic groups (Yoruba and Igbo), and Southern ethnic minorities. The prevalence of girl-child marriage was determined for the five ethnic groups and individually for each ethnic minority group. Relationships between ethnicity and religious affiliation on girl-child marriage were explored using Cox proportional hazard regression models, adjusting for residence, education and wealth quintile.

**Results:**

Child marriage was higher for the Northern majority ethnic group of Hausa/Fulani (54.8%) compared to the two major Southern ethnic groups (3.0–3.6%) and aggregated Northern ethnic minorities (25.7%) and Southern minorities (5.9%). However, overall, the less known Northern ethnic minority groups of Kambari (74.9%) and Fulfude (73.8%) recorded the highest prevalence. Compared to the major Southern ethnic group of Yoruba, the adjusted hazard ratio (AHR) of child marriage was significantly higher for Northern ethnic minorities (AHR = 2.50; 95% C.I. = 1.59–3.95) and Northern major ethnicity (AHR = 3.67, 95% C.I. = 2.33–5.77). No significant difference was recorded among Southern ethnic groups. Girls affiliated to other religions (Muslim and traditionalist) had higher child-marriage risks compared to Christians (AHR = 2.10; 95% C.I. = 1.54–2.86).

**Conclusion:**

Ethnicity and religion have independent associations with girl-child marriage in Nigeria; interventions must address culturally-laden social norms that vary by ethnic groups as well as religious-related beliefs.

## Background

Globally 39,000 girls under the age of 18 years are married daily and 14.2 million girls annually [1]. More than 700 million women alive worldwide were married before age 18 [2]. The highest prevalence of girl-child marriage is in South Asia and Sub-Saharan Africa [2]. Early marriage violates the human rights of the girl-child, increases her risk of maternal morbidity and mortality, and robs her of educational and developmental opportunities [3–5]. There is a global consensus to end girl-child marriage and Target 5.3 of the Sustainable Development Goals (SDGs) is to “eliminate all harmful practices, such as child, early and forced marriage and female genital mutilations” by 2030 [6]. Ending girl-child marriage has the potential to contribute to eight SDGs, including those addressing poverty (goal 1), good health and well-being (goal 3), inclusive and quality education (goal 4), gender equality (goal 5), and economic growth (goal 8) [7, 8]. Progress in reducing child marriage rate has so far been quite slow in sub-Saharan Africa [2, 9].

Nigeria, with over 3.5 million under-18 girls currently married [10] has the highest number of child brides in Africa [7, 10] and the third highest number in the world [8]. The rate of child marriage varies significantly by geo-political zones in Nigeria, ranging from 39.0% to 67.6% for the Northern zones compared to the much lower rates of 13.9-21.6% for the Southern zones [11]. Girl-child marriage rate in Nigeria has not improved over the years with only a 1% decline in over three decades. Whereas Nigeria passed the Child Rights Act that prohibits marriage below the age of 18 in 2003, 12 Northern states (out of 36 states) are yet to domesticate the Act. If the current pattern continues, Nigeria’s population of child bride is expected to double by 2050 [2].

Globally, child marriage is associated with inequitable gender norms, which are deeply engrained in local socio-cultural context [12, 13] and associated with poverty, low educational level and rural location [14–22]. Surprisingly, very few peer-reviewed research has been published on child marriage in Nigeria [12–19] and most are small-scale studies lacking rigorous analysis of the determinants. In particular, very few studies on girl-child marriage have examined the role of ethnicity – a representation of local practices/values and a sociological marker of cultural diversities [23, 24].

Ethnicity is particularly important in the context of Nigeria – a nation with 374 identifiable ethnic groups [25] with substantial variation in ethnic cultural beliefs and practices. Nigeria’s ethnic groups include the three major ones – Hausa (30%) concentrated in the North, Yoruba (15.5%) in the South-West, Igbo (15.2%) in the South-East, and several ethnic minority groups – Fulani (6%), Tiv (2.4%), Kanuri/Beriberi (2.4%) in the North, and Ibibio (1.8%), Ijaw (1.8%) in the South-South, and many others accounting for 24.7% [26]. In Nigeria, ethnicity has remained a major underlying factor associated with many health-related and social behaviours, including risky sexual behaviour, poor contraceptive uptake or discontinuation of use, poor maternal healthcare utilization, female genital mutilation, intimate partner’s violence and so many others [27–32]. Despite being an important sociological marker of cultural diversities, the role of ethnicity on child marriage has not been sufficiently ascertained in empirical studies in Nigeria. Interplaying with religious beliefs, the multi-ethnic setting of the country serves as a predisposition for varied marital ideologies that tend to portend health risk for a girl-child.

Nigeria is also a multi-religious society with three major divisions – Islam (53.5%), Christianity (45.9%), adherents of Nigeria’s indigenous religions and others (6.0%) [26]. Religious beliefs have a significant role in shaping gender-related behaviours and practices. Nigeria’s geo-political zones, interestingly, are characterised by an interplay of religion and cultural values; religion may be associated with the sociocultural framing of gender norms and girl-child marriage practices [33, 34]. Though not limited to one religious group, available evidence reveals that child marriage is more prevalent in Muslim communities [35, 36]. This is associated with the belief among the conservative Muslims that Quran allows girl’s marriage at any age, and Prophet Muhammad’s wife was nine years at marriage [34]. In Nigeria, the Northern States refusing domestication of Nigeria’s Child Right Act are Muslim dominated and are reacting to protect their ethno-religious standpoint on child marriage. There are however a contrary perspective by other Muslims who argue that a girl-child should attain puberty and emotional maturity before marriage [35].

Though higher level of education, socioeconomic status, and urban residence are precursors for reducing child marriage practice [14, 18, 36], variations subsist due to ethnic and religious differences. For example, in Malaysia, despite the country’s economic progress and high educational level, child marriage practice remains high and is underpinned in religious and traditional beliefs [35]. In Nigeria, educated Hausa/Fulani girls may not have the same risk of child marriage as educated Yoruba or Igbo counterparts. However, there is need for empirical evidence indicating whether educated adolescents or those who share some other specific socioeconomic characteristics have the same risk of child marriage across ethnic and religious affiliations in Nigeria.

To date, the available peer-reviewed publications have hardly rigorously examined and explained, either singly or jointly, the influence of ethnic and religious beliefs on girl-child marriage in Nigeria. This study aims to address the existing research gap by specifically examining the influence of ethnic and religious affiliations on girl-child marriage based on a nationally representative sample of female adolescents in Nigeria. The empirical evidence generated will contribute to a better understanding of the dynamics of girl-child marriage in multi-religious and multi-ethnic society settings and can inform better framing and contextualisation of interventions.

## Methods

### Sample design

This study utilized the Nigeria Demographic and Health Survey (NDHS) dataset of 2013, which was the latest NDHS dataset available at the time of this research. For this study, approval was received from the ICF International to utilize the NDHS data. The NDHS collects demographic and health indicators from a nationally representative sample of men and women of reproductive age across Nigeria’s 36 states. The 2013 NDHS involved 904 clusters, from which samples were selected using stratified two-stage cluster design, and data collected from 38,948 women aged 15–49 years. For this study, data of females age 15–19 with a weighted sample size of 7804 were analysed. The sample weight, which is a correction factor to adjust for variations in selection probability in the dataset, was calculated by multiplying the household weight by the inverse of the individual’s share of her individual response rate group. The detail is published in the NDHS report [37]. The weight was applied to the descriptive analysis using “[iw = weight]”, while the Chi-squared test and survival analysis were adjusted for the weighted complex survey design using the “svy: tab” and “svy: stcox” Stata commands respectively.

Research on child marriage has largely focused on either age 20–24 years [22, 38, 39] or age 15–19 years [40]. Using age 20–24 carries the risk that the socioeconomic condition between the age at marriage and the current age of some respondents might have changed substantially, and this is particularly true of religious affiliation in Nigeria’s multi-religious setting. Using age 15–19 years, on the other hand, focuses on those currently at risk of girl-child marriage but tends to under-report the level of child marriage as those unmarried and younger than age 18 during the survey may still marry before age 18. We focussed on girls aged 15–19 whose exposure to child marriage risk is more recent and can be matched with their current socioeconomic condition. Further, we focussed on the age group because of the higher priority we accorded to assessing the independent roles of ethnicity and religious affiliations in girl-child marriage as our foremost objective.

### Variables measurements

The outcome variable for this study is child marriage, defined as marriage before age 18 [40]. The variable was generated using respondents’ self-reported marital status and age at marriage. Respondents were categorized based on marital status and age at marriage: those married before age 18 were categorized as child brides. The key explanatory variables are ethnicity and religious affiliation. All the Nigerian ethnic groups in the dataset were grouped into five, including the three major ethnic groups – Hausa/Fulani, Igbo and Yoruba, and the ethnic minority groups which were categorised into two: Northern ethnic minority and Southern ethnic minority groups. Hausa and Fulani are the most similar ethnic groups in Nigeria, thus are often grouped together [27–31]. The ethnic minority groups were categorised based on the North-South divide of the country and geographical proximity of the ethnic groups in the North and South as observed by Okunogbe [41]. Besides, ethnic minority groups in the North and those in the South were grouped separately because of our consideration for religious affiliation – those in the North are mostly Muslims while their counterparts in the South are mainly Christians. Operational definitions and geographical distributions for these and other selected independent variables are presented in Additional file 1. Other independent variables were treated as potential confounding variables. The selection of covariates was guided by extant literature.

### Statistical analyses

Univariate analysis was undertaken for socio-demographic characteristics and the prevalence of child marriage generated for the five categories of ethnic groups. In addition, we generated girl-child marriage prevalence individually for all ethnic tribes with at least 50 participants in the sample. Pearson’s Chi-squared test of independence was used to determine bivariate associations between child marriage and selected independent variables. Kaplan-Meier survival estimates curves were used to depict the risk of child marriage among ethnic groups and religious affiliations. Cox proportional hazard model was used to determine the girls’ risk of child marriage in relation to ethnic and religious affiliations in recognition of the censored nature of the data relating to child marriage in the study population. Censoring is problematic to time-ordered data like marriage among adolescents because the entire lifespan of child marriage event was not covered for those aged below 18 years and not yet married at the time of the survey. Since we were not certain of their timing of marriage, these observations were right censored. Cox regression permits modelling of the censored time-ordered data as the dependent variable, assuming that the effect of covariates on hazard rates is exponential [40, 42]. In our analysis, married adolescents were the non-censored cases while those still single were the right-censored observations.

The probability of girl-child marriage is the hazard, and this was modelled using the following equation (Eq.):

1 $$ H(t)={H}_0(t)\ {e}^{\left({b}_1{X}_1+{b}_2{X}_2+{b}_3{X}_3+\dots +{b}_k{X}_k\right)} $$

Where X_1_ … X_k_ are a set of explanatory variables and H_0_(t) is the baseline hazard at time t, representing the hazard for a person with the value 0 for all the explanatory variables. When we divide both sides of Eq. (1) by H_0_(t) and taking logarithms, Eq. (1) becomes:

2 $$ \ln \left[\frac{H(t)}{H_0(t)}\right]={b}_1{X}_1+{b}_2{X}_2+{b}_3{X}_3+\dots +{b}_k{X}_k $$

Where H(t)/H_0_(t) is the hazard ratio (HR). The coefficients b_1_ … b_k_ are estimated by Cox regression [27, 43].

Two models were fitted in multivariate analysis: While model 1 examined the unadjusted effects, models 2 examined the effects of ethnic and religious affiliations adjusted for education, residence and wealth quintile. The analyses were conducted using Stata software (version 15.1) and statistical significance determined at 95% confidence level for the hazard ratio.

## Results

### Socio-demographic characteristics of the study population

About three-fifths (62%) of our sample of 7804 girls aged 15–19 years were below age 18 (Additional file 1). The ethnic distribution is as follows: Hausa/Fulani (35%), Northern ethnic minorities (24%), Southern ethnic minorities (14%), Igbos (14%) and Yoruba (13%) (Additional file 1). The Muslims constituted 52.6%, Christians 46.5%, and the traditional religion 0.9%. Majority of the respondents were rural-dwellers (58%), had secondary or higher education (60%) and were between the poorest and middle wealth quintiles (58%).

**Table 1:** Bivariate association between child marriage and ethnicity, region of residence and socioeconomic characteristics of female adolescents in Nigeria

	Child marriage experience			
Ethnicity, religion and socioeconomic characteristics	Child marriage (*n* = 2118)	Others (*n* = 5686)	Total (*N* = 7804)	Chi-Squared
	n (%)	n (%)	N	
Ethnicity				
Hausa/Fulani	1496 (54.8)	1236 (45.2)	2732	159.15***
Igbo	39 (3.6)	1031 (96.4)	1070	
Yoruba	31 (3.0)	979 (97.0)	1010	
^a^Southern ethnic minorities	64 (5.9)	1026 (94.1)	1091	
^b^Northern ethnic minorities	488 (25.7)	1414 (74.3)	1902	
Religion				
Christianity	234 (6.5)	3384 (93.5)	3618	237.29***
Islam	1856 (45.5)	2228 (54.5)	4084	
Traditional	15 (23.0)	52 (77.0)	67	
Level of Education				
No/Primary	1834 (58.8)	1283 (41.2)	3117	1075.76***
Secondary/higher	284 (6.1)	4403 (93.9)	4687	
Place of residence				
Rural	1822 (40.5)	2682 (59.5)	4504	200.12***
Urban	296 (9.0)	3004 (91.0)	3300	
^h^Wealth quintile				
Poorest	838 (63.5)	482 (36.5)	1320	274.41***
Poorer	689 (43.8)	883 (56.2)	1571	
Middle	349 (21.3)	1292 (78.7)	1641	
Richer	191 (11.5)	1466 (88.5)	1657	
Richest	51 (3.2)	1564 (96.8)	1615	
**Total**	**27.1**	**72.9**	**7804**	

Note: respondents who married before age 18 were categorized as child marriage while those who were still unmarried or married at age 18 or above at the time of the survey were categorized as others

^a^Combination of ethnic minorities in Southern Nigeria; ^b^combination of ethnic minorities in Northern Nigeria; ^h^household wealth quintile

*** *p* < .001

### Girl-child marriage prevalence and bivariate analysis of associated factors

Twenty-seven percent of the respondents experienced child marriage (Table 1). Among the six main ethnic groupings, Hausa/Fulani girls (54.8%) had the highest prevalence of girl-child marriage, followed by the Northern ethnic minorities (25.7%) while the lowest rates were among the major Southern tribes (Yoruba – 3.0%; Igbo – 3.6%). Detailed analysis of ethnic minority groups (Additional file 1) showed that two Northern ethnic minority groups of Kambari (74.9%) and Fulfude (73.8%) recorded the highest girl-child marriage in Nigeria – higher than the rate for the Hausa/Fulani. Child marriage prevalence was higher among girls who were Muslims (46%) compared to those of other religions (Christians – 7%, traditionalists – 23%). Both ethnicity and religious affiliations were significantly associated with girl-child marriage at the bivariate levels. Education, location (urban/rural) and wealth status were also significantly associated with girl-child marriage at the bivariate level.

**Fig. 1:**
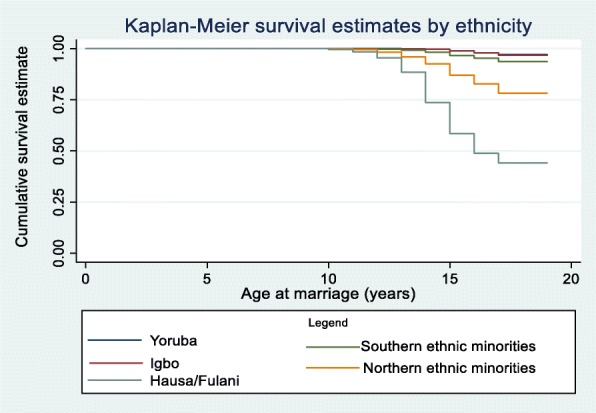
Child marriage survival curve by ethnicity for a representative sample of female adolescents in Nigeria

**Fig. 2:**
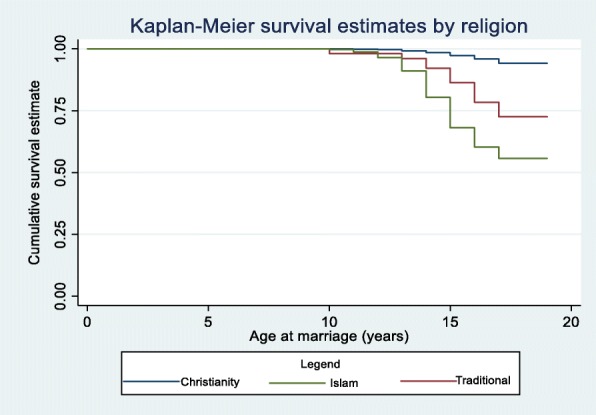
Child marriage survival curve by religious affiliations for a representative sample of female adolescents in Nigeria

As the Kaplan-Meier survival estimates show, Hausa/Fulani girls married much earlier compared to other ethnic groups: About 99% of those of Igbo and Yoruba ethnic groups, 90% of the female adolescents of Southern ethnic minorities, 80% of those in the Northern ethnic minorities were unmarried by age 18 compared to about 50% of the Hausa/Fulani ethnic group (Fig. 1). Christian girls have a higher rate of not experiencing early marriage compared to their peers with Muslim or traditional religion affiliation: by age 18, about 55% of Muslim girls and 70% of traditional religion practitioners were unmarried compared to more than 90% of Christians (Fig. 2).

### Multivariate analysis of the risk of girl-child marriage by ethnicity and religious affiliations

The unadjusted hazard ratio (UHR) results (model 1) of the Cox proportional hazard analysis (Table 2) shows a significant association between the risk of child marriage and both ethnicity and religion (*p* < 0.05). Compared to Yoruba girls, the risk of child marriage was about two-fold higher for girls of Southern ethnic minorities (UHR = 1.97; p < 0.05; 95% C. I = 1.19–3.26), nine-fold higher for girls of Northern ethnic minorities (UHR = 9.35; p < 0.05; 95% C.I. = 5.91–14.80) and 24-fold higher for girls of Hausa/Fulani ethnic background (UHR = 24.44; p < 0.05; 95% C.I. = 15.90–37.56). However, the risk of child marriage was not significantly different between the two major Southern ethnic groups (Yoruba and Igbo). Compared to the Christians, girls of other religions (Muslim and traditionalist) had about nine-fold higher risk of child marriage (UHR = 8.68; 95% C.I. = 6.80–11.09).

**Table 2:** Cox proportional hazard model showing the effects of ethnicity and religious affiliation on child marriage among female adolescents in Nigeria, adjusting for level of education, place of residence and wealth quintile

Ethnicity, religion and socioeconomic characteristics	Model 1			Model 2		
	UHR	SE	(95% C.I.)	AHR	SE	95% C.I.
**Ethnicity**						
Yoruba^*RC*^	1.00			1.00		
Igbo	1.20	0.34	0.69–2.08	1.53	0.46	0.85–2.76
Southern ethnic minorities	1.97 **	0.51	1.19–3.26	1.49	0.42	0.86–2.60
Northern ethnic minorities	9.35***	2.19	5.91–14.80	2.50***	0.58	1.59–3.95
Hausa/Fulani	24.44***	5.35	15.90–37.56	3.67***	0.85	2.33–5.77
**Religion**						
Christianity^*RC*^	1.00			1.00		
Others (Islam & Traditional)	8.68***	1.08	6.80–11.09	2.10***	0.33	1.54–2.86
**Level of Education**						
No/Primary^*RC*^				1.00		
Secondary/Higher				0.22***	0.02	0.19–0.27
**Place of residence**						
Rural^*RC*^				1.00		
Urban				0.51***	0.06	0.40–0.64
**Wealth quintile**						
Poorest^*RC*^				1.00		
Poorer				0.96	0.08	0.81–1.13
Middle				0.86	0.09	0.70–1.05
Richer				0.94	0.12	0.72–1.21
Richest				0.48***	0.10	0.32–0.72

Note: 95% confidence intervals (C.I.) are presented in parenthesis. UHR = unadjusted hazard ratio; AHR = adjusted hazard ratio (adjusting for level of education, place of residence and wealth quintile); SE = Standard error; C.I. = Confidence Interval; ^RC^reference category

*** *p* < .001. ** *p* < .01

Adjusting for the place of residence, education and wealth quintile, the pattern of association between ethnicity and child marriage remains fairly consistent. The adjusted hazard ratio (AHR) of child marriage was significantly higher among girls of Northern ethnic minorities (AHR = 2.50; 95% C.I. = 1.59–3.95) and the Northern major ethnic group of Hausa/Fulani (AHR = 3.67, 95% C.I. = 2.33–5.77) compared to Yoruba ethnic group but no significant difference between Yoruba and Igbo. However, unlike the findings of the unadjusted ratios, no significant difference was recorded between girls of the major Southern tribe of Yoruba and Southern minority tribes. Girls with other religions (Muslim and traditionalist) had a two-fold adjusted higher risk compared to Christian girls (AHR = 2.10; 95% C.I. = 1.54–2.86). Ethnicity and religious affiliations remain significant in further multivariate analysis for girls in similar socioeconomic contexts in terms of higher educational level, urban residence, and high wealth quintile (Table 3).

**Table 3:** Cox proportional hazard model of effect of ethnic and religious variations on child marriage among adolescents with similar level of education, residence and wealth quintile

Ethnicity, religion by same level of education, residence and wealth quintile	Model 1			Model 2		
	UHR	SE	95% C.I.	AHR	SE	95% C.I.
**Risk among adolescents with secondary/higher education**						
***Ethnicity***						
Yoruba ^*RC*^	1.00			1.00		
Igbo	1.56	0.51	0.82–2.96	1.85	0.66	0.82–2.96
Southern ethnic minorities	1.88*	0.57	1.04–3.40	2.22*	0.72	1.17–4.15
Northern ethnic minorities	3.99***	1.12	2.30–6.91	4.14***	1.17	2.35–7.07
Hausa/Fulani	7.22***	1.99	4.19–12.43	5.94***	1.73	3.27–10.25
***Religion***						
Christianity ^*RC*^	1.00			1.00		
Others (Islam & Traditional)	2.54***	0.38	1.89–3.42	1.44	0.33	0.93–2.27
**Risk among adolescents in urban residence**						
***Ethnicity***						
Yoruba ^*RC*^	1.00			1.00		
Igbo	1.29	0.48	0.62–2.66	2.47*	1.05	1.07–5.69
Southern ethnic minorities	1.35**	0.59	0.57–3.20	2.36	1.08	0.96–5.83
Northern ethnic minorities	6.58***	2.28	3.33–13.03	5.72***	1.89	2.98–10.97
Hausa/Fulani	9.24***	2.94	4.94–17.27	5.74***	1.87	3.02–10.91
***Religion***						
Christianity ^*RC*^	1.00			1.00		
Others (Islam & Traditional)	5.66***	1.19	3.75–8.55	3.09***	0.88	1.76–5.43
**Risk among adolescents with middle/higher wealth index**						
***Ethnicity***						
Yoruba ^*RC*^	1.00			1.00		
Igbo	1.20	0.36	0.66–2.17	1.94	1.00	0.99–3.79
Southern ethnic minorities	1.88*	0.53	1.08–3.27	2.94**	0.91	1.59–5.41
Northern ethnic minorities	6.17***	1.60	3.70–10.28	6.11***	1.59	3.67–10.19
Hausa/Fulani	13.10***	3.39	7.89–21.77	8.88***	2.36	5.27–14.95
***Religion***						
Christianity ^*RC*^	1.00			1.00		
Others (Islam & Traditional)	5.17***	0.76	3.87–6.91	2.40***	0.51	1.58–3.64

Note: Table focuses only on adolescents with the same level of education, residence and wealth quintile across Nigeria. UHR = unadjusted hazard ratio (Model 1); AHR = adjusted hazard ratio, adjusting between ethnicity and religion (Model 2); SE = Standard error; C.I. = confidence intervals. ^RC^reference category

*** *p* < .001. ** *p* < .01. * *p* < .05

## Discussion

Girl-child marriage is a leading adolescent health and development concern of global importance but so far under-researched in Nigeria despite the country’s very high burden of child marriage [10]. This study is one of the few that has so far examined some determinants of girl-child marriage in Nigeria using a nationally representative sample. We have specifically focussed on the role of ethnicity and religious affiliation – two key elements in the socio-cultural dynamics relating to girl-child marriage, but which had been largely unaddressed in previous research.

We found that girl-child marriage prevalence differs considerably among girls aged 15–19 years from different ethnic groups in Nigeria. On the one hand, we found the level of girl-child marriage among the Hausa/Fulani – the major Northern ethnic group – to be about 15–18 times higher than that of the Southern major ethnic groups (Yoruba and Igbo). This finding accords with previous reports of a higher girl-child marriage rate in Northern Nigeria compared to the South [14, 25, 42] and among the Hausa/Fulani compared to other major tribes [11, 15]. The high rate of girl-child marriage among the Hausa/Fulani is promoted, among others, by parents’ betrothal of their daughters in order to sustain family alliance, seal up friendship, fulfil a promise or appreciate their benefactor [44]. This cultural practice permits parents or guardian to betroth a girl-child – usually from childhood or at puberty age without her consent – to an individual who had previously provided financial or otherwise support to the family. Contrariwise, such practice is less pronounced among the major southern ethnic groups, thus the low prevalence of child marriage in the southern region.

On the other hand, contrary to what Adebowale [15] had reported in the only published work that used a nationally representative sample to examine the relationship between ethnicity and girl-child marriage, we found that the Hausa/Fulani group does not actually have the highest girl-child marriage in Nigeria. The ethnic groups with the highest rates are the Kambari and Fulfude – two Northern ethnic minority groups with prevalence of 74.9% and 74.8% respectively compared to 54.8% among the Hausa/Fulani group. This finding support the claim that regardless of geographical co-existence, ethnic groups in Nigeria are distinct and diverse in beliefs and practices, particularly in relation to gender norms [41]. To the best of our knowledge, this is the first study to report the prevalence of girl-child marriage individually for each minority tribe, unlike previous studies [14, 15, 25, 42]. Also, unlike our more rigorous approach of classifying ethnic groups in the North and South separately, Adebowale [14], following the approach that has been used in other studies on ethnicity and health outcomes in Nigeria, had lumped all ethnic minorities in the country into one [27–30, 45]. This popular approach obviously and sadly results in a lost opportunity to uncover any difference that may exist between geographically-, culturally-, and religiously-diverse ethnic minority groups and/or between the minority and majority ethnic groups co-existing within the same geographical regions in Nigeria.

Secondly, we found that both ethnicity and religion have significant independent influences on girl-child marriage practice in Nigeria. Specifically, our result shows that not only are there considerable differences between the major tribe of the North (the Hausa/Fulani) and those of the South (Yoruba and Igbo), but considerable differences also occur between major and minor ethnic groups in both the Northern and the Southern part of Nigeria – a result that is made possible by our more rigorous and appropriate approach to ethnic group classification. To the best of our knowledge, this study is the first to provide girl-child marriage prevalence figures specifically and separately for the Southern and Northern ethnic minority groups and to document the intra-regional differences between the majority and the minority ethnic groups. Despite Nigeria’s ethnic diversities, most studies on child marriage have focused on either only one ethnicity or the three major ethnic groups – Hausa, Igbo, Yoruba [46], while other studies considered regions of residence [14] which are often ethnically heterogeneous. Some studies combined all the ethnic minority groups as ‘others’ [15], albeit they are culturally and geographically diverse. The present study attempted to address the deficiencies identified in ethnic categorizations in previous studies. We took Nigeria’s religious and cultural diversities into consideration by not categorizing all minority ethnic groups as one in our ethnic grouping.

The significant differences in the prevalence of girl-child marriage among the ethnic groups reflect differences in traditional beliefs, cultural values and social norms that relate not only to girl-child marriage but also to the broader issue of the perceived value of females and the degree of women’s autonomy. The strong desire to prevent young girls from engaging in premarital sex, which reflects the high value placed on girls’ pre-marriage virginity status and the strong stigma associated with premarital pregnancy and its implications for the family’s honour in traditional societies, have featured prominently in the cultural framing of the girl-child marriage. Viewed broadly, these issues are all rooted in an entrenched system of gender inequality and inequitable social norms that particularly plays out in traditional patriarchal societies and drive the age-long practice of child marriage [12, 13, 47–49].

The influence of religious affiliation as an independent factor in child marriage practice with Muslim girls having a higher rate than Christians is worth noting particularly in the Nigerian context where religion plays a significant role in shaping values and practices at individual, household and community level, including girl-child development issues [50]. It is interesting, for example, to note that the 12 states with the highest prevalence of girl-child marriage in Nigeria are all Muslim-dominated states that have instituted Sharia laws and have so far refused to domesticate the Child Rights Act [34]. This picture reflects a classical interplay of religion and culture. As Braimah highlighted, “The Hausa-Fulanis in Northern Nigeria show strict adherence to the Quran and the Prophet Muhammad’s Sunnah. As Islam plays a pivotal role in the lives of most Northern Nigerians, the culture and traditions of the Hausa-Fulanis are intertwined with the Islamic religion. Therefore, due to the influence of Islam and Muhammad’s marriage to Aisha, as reported in the Hadiths, it is not surprising that Muslims in Northern Nigeria endorse and practise child marriage” [34]. The widely publicized case of girl-child marriage involving a one-time state governor and later a senator, Ahmad Yerima, who married a 13-year-old Egyptian girl in 2010 provides a clear illustration regarding power of religion in driving and sustaining the practice of girl-child marriage. In his response to the public outcry over his behaviour, Senator Yerima justified his marriage on religious grounds, stating that he was simply following the teaching and practice of Prophet Muhammad who married Aisha at the age of nine [34].

While the use of a nationally representative sample is one of the key strengths of this study, the use of secondary data also has its drawback as it constrains analysis to the variables with already collected information, which limits deeper exploration of germane issues such as cultural values and sociocultural framing of gender inequality that underlie girl-child marriage. Future studies should complement secondary quantitative data analysis with qualitative data for further interrogation and understanding of the sociological dynamics relating to girl-child marriage. Also, this study analysed self-reported information with the limitation of lack of any mechanism for validating the responses provided and reported. Furthermore, since this study is based on cross-sectional data, we can only infer association but in no way can we imply causality.

## Conclusion

Overall, our findings strongly suggest that to be effective, interventions aimed at reducing girl-child marriage in Nigeria must give considerable attention to culturally-laden social norms that vary by ethnic groups as well as religious-related beliefs. Among others, efforts must include working closely with community structures and stakeholders in designing and implementing context-specific and culturally-relevant interventions, use of effective social and behavioural communication strategies at community levels to address gender issues and social norms, and active engagement with influential traditional and religious leaders who can champion the fight against girl-child marriage. Targeted approaches to specific ethnic groups with a high level of prevalence as identified in the study may provide a good starting point towards achieving Nigeria’s new National Strategy to End Child marriage, with its vision of reducing child marriage by 40% by 2020 and ending the practice entirely by 2030.

## Supplementary information

**Additional file 1: Appendix 1**. Independent variables for modeling girl-child marriage in Nigeria. **Appendix 2**. Distribution of the study sample by socio-demographic and socioeconomic characteristics. **Appendix 3**. Prevalence of child marriage among adolescents in various ethnic groups by secondary or higher education, urban residence and middle to upper wealth quintile.

## Data Availability

The datasets generated and analysed during the current study are available in the Demographic and Health Survey (DHS) program repository, www.dhsprogram.com

## Abbreviations

AHR: Adjusted Hazard Ratio
C.I.: Confidence Interval
MICS: Multiple Indicator Cluster Survey
NDHS: Nigeria Demographic and Health Survey
NPC: National Population Commission Nigeria
SDG: Sustainable Development
SE: Standard Error
UNICEF: United Nations Children’s Fund
UNDP: United Nations Development Programme
UHR: Unadjusted Hazard Ratio
WHO: World Health Organization
