# Supplementary material for: Ethnicity, religious affiliation and girl-child marriage: a cross-sectional study of nationally representative sample of female adolescents in Nigeria
Source: BMC Public Health. 2020 Apr 29;20:583. doi: 10.1186/s12889-020-08714-5 (PMC7189516; doi:10.1186/s12889-020-08714-5)
Supplement: Supplementary file 1 — Additional file 1: Appendix 1. Independent variables for modeling girl-child marriage in Nigeria. Appendix 2. Distribution of the study sample by socio-demographic and socioeconomic characteristics. Appendix 3. Prevalence of child marriage among adolescents in various ethnic groups by secondary or higher education, urban residence and middle to upper wealth quintile. [file 12889_2020_8714_MOESM1_ESM.docx]

**Appendix 1.** Independent variables for modeling girl-child marriage in Nigeria

| **Variables** | **Operational Definitions** |
| --- | --- |
| Ethnic affiliation | All the eligible respondents were grouped into five ethnic classifications: Hausa/Fulani, Igbo, Yoruba, Northern ethnic minorities and Southern ethnic minorities. The last two ethnic categories embrace all the other (minority) ethnic groups in Northern and Southern Nigeria respectively, each of which has below 5% of the Nigerian population. Though each ethnic group spreads across the country, the Hausa/Fulani ethnic group are concentrated in the Northern part of Nigeria, the Igbos are located in the South-East while the Yorubas are concentrated in the South West. While all the Northern ethnic minorities are in the North (North Central, North East and North West), the Southern ethnic minorities are concentrated in the South-South. |
| Religious affiliation | Religious affiliation was grouped into Christians, Muslim and traditional groups. In multivariate analsyis, this was further ollapsed into two (i) Christians, and (ii) traditional and Muslim, given the low number of traditional religious practioners in the sample. While The Hausa/Fulani ethnic group were about 99% Muslim and Igbo are 99% Christians, the Yorubas have nearly equal proportions of Muslim and Christians with about 5% Traditionalist. |
| Age of respondent | Self-reported age of respondent at time of survey: 15, 16, 17, 18, 19 |
| Education | Respondent’s highest level of education attained: no formal education, primary, secondary, higher. For ease of interpretation and clarity of association with child marriage, educational level, this was further collapsed into two in multivariate analsyis: secondary/higher and below secondary education |
| Wealth index | Composite index of household items/amenities, electrical appliances, toilet facility, drinking water, and floor/wall materials grouped into a quintile: poorest, poorer, middle, richer, richest |
| Place of residence | Respodent’s place of residence at time of survey: urban, rural |

**Appendix 2: Distribution of the study sample by socio-demographic and socioeconomic characteristics**

| Socio-demographic and socioeconomic characteristics | Number of adolescents | Proportions (%) |
| --- | --- | --- |
| **Age (years)** |  |  |
| 15 | 2014 | 25.8 |
| 16 | 1464 | 18.8 |
| 17 | 1378 | 17.7 |
| 18 | 1784 | 22.9 |
| 19 | 1164 | 14.9 |
| **Level of Education** |  |  |
| No formal education | 2165 | 27.7 |
| Primary | 952 | 12.2 |
| Secondary | 4562 | 58.5 |
| Higher | 125 | 1.6 |
| **Place of residence** |  |  |
| Urban | 3300 | 42.3 |
| Rural | 4504 | 57.7 |
| **Wealth quintile** |  |  |
| Poorest | 1320 | 16.9 |
| Poorer | 1571 | 20.1 |
| Middle | 1641 | 21.0 |
| Richer | 1657 | 21.2 |
| Richest | 1615 | 20.7 |
| **Religion** ^c^ |  |  |
| Christianity | 3618 | 46.6 |
| Islam | 4084 | 52.5 |
| Traditional | 67 | 0.9 |
| **Ethnicity** |  |  |
| Hausa/Fulani | 2732 | 35.0 |
| Igbo | 1070 | 13.7 |
| Yoruba | 1010 | 12.9 |
| ^a^Southern ethnic minorities | 1091 | 14.0 |
| ^b^Northern ethnic minorities | 1901 | 24.4 |
| **Total** | **7,804** | **100.0** |

^a^other ethnic groups in Northern Nigeria with small populations each; ^b^other ethnic groups in South-Southern region of Nigeria; ^c^missing values were excluded

**Appendix 3: Prevalence of child marriage among adolescents in various ethnic groups by secondary or higher education, urban residence and middle to upper wealth quintile**

| Ethnicity | Number of adolescents | Prevalence in each ethnic group | Prevalence among those with secondary/ higher education | | Prevalence among those living in urban areas | | Prevalence among those with middle or upper wealth quintile | |
| --- | --- | --- | --- | --- | --- | --- | --- | --- |
|  | N (%)^c^ | n (%)^d^ | % | n^e^ | % | n^f^ | % | n^g^ |
| **Nigeria** | **7804 (100.0)** | **2118 (27.1)** | **6.1** | **4,687** | **9.0** | **3,300** | **21.1** | **1,641** |
| **Major ethnic groups** |  |  |  |  |  |  |  |  |
| Hausa/Fulani | 2,732 (35.0) | 1,496 (54.8) | 14.3 | 761 | 20.2 | 816 | 30.8 | 1,043 |
| Igbo | 1,070 (13.7) | 39 (3.6) | 3.3 | 980 | 3.1 | 792 | 3.3 | 892 |
| Yoruba | 1,010 (12.9) | 31 (3.0) | 2.1 | 953 | 2.4 | 772 | 2.7 | 972 |
|  |  |  |  |  |  |  |  |  |
| **Southern ethnic minorities** | **1091 (14.0)** | **64 (5.9)** | **3.9** | **925** | **3.2** | **424** | **5.1** | **956** |
| Ijaw/Izon | 173 (2.2) | 17 (9.8) | 8.4 | 144 | 9.2 | 60 | 11.0 | 151 |
| Ibibio | 166 (2.1) | 7 (4.1) | 4.1 | 155 | 0.0 | 43 | 4.7 | 147 |
| Urhobo | 107 (1.4) | 4 (3.4) | 2.4 | 88 | 4.0 | 45 | 3.8 | 97 |
| Bini/Edo | 65 (0.8) | 2 (2.6) | 1.4 | 61 | 1.7 | 51 | 2.7 | 62 |
| Annang | 52 (0.7) | 0 (0.0) | 0.8 | 45 | 0.0 | 7 | 0.8 | 48 |
| Ogoni | 60 (0.8) | 0 (0.0) | 0.0 | 54 | 0.0 | 6 | 0.0 | 59 |
| Others^a^ | 468 (6.0) | 28 (6.5) | 3.9 | 361 | 2.7 | 203 | 5.0 | 376 |
|  |  |  |  |  |  |  |  |  |
| **Northern ethnic minorities** | **1901 (24.4)** | **488 (25.7)** | **8.1** | **1,068** | **15.0** | **495** | **15.8** | **1,050** |
| Igala | 103 (1.3) | 15 (14.8) | 13.8 | 86 | 15.4 | 42 | 15.2 | 87 |
| Kambari | 106 (1.4) | 79 (74.9) | 0.0 | 4 | 0.0 | 4 | 32.0 | 11 |
| Kanuri/Beriberi | 146 (1.9) | 58 (39.8) | 11.5 | 42 | 24.2 | 80 | 26.7 | 64 |
| Nupe | 121 (1.6) | 23 (18.8) | 3.1 | 73 | 11.9 | 45 | 20.3 | 113 |
| Tiv | 175 (2.2) | 48 (27.2) | 8.0 | 91 | 4.6 | 19 | 15.2 | 67 |
| Fulfude | 68 (0.9) | 50 (73.8) | 31.0 | 10 | 37.1 | 19 | 46.7 | 16 |
| Idoma | 77 (1.0) | 7 (9.1) | 5.3 | 61 | 0.0 | 29 | 7.3 | 59 |
| Ebira/Igbira | 70 (0.9) | 9 (12.2) | 10.8 | 62 | 15.3 | 27 | 12.3 | 69 |
| Others^b^ | 1,036 (13.2) | 206 (19.3) | 7.3 | 654 | 12.9 | 238 | 13.7 | 579 |
| **Religion** ^h^ |  |  |  |  |  |  |  |  |
| Christianity | 3618 (46.5) | 234 (6.5) | 4.1 | 3,116 | 3.1 | 1,828 | 4.8 | 2,912 |
| Islam | 4084 (52.6) | 1856 (45.5) | 10.2 | 1,524 | 16.3 | 1,453 | 23.1 | 449 |
| Traditional | 67 (0.9) | 15 (23.0) | 1.0 | 27 | 41.5 | 4 | 0.8 | 33 |

^a^Other ethnic groups in Southern Nigeria whose sample are below 50 each; ^b^Other ethnic groups in Northern Nigeria whose samples are below 50 each; ^c^Percentage distribution of the sampled adolescents in each ethnic group; ^d^Percentage of child marriage among adolescents in each ethnic group; ^e^Total sample of adolescents with secondary/higher education; ^f^Total sample of adolescents living in urban area; ^g^Total sample of adolescents with middle or upper wealth quintile; ^h^Missing values excluded.
